# The interaction between cortisol and testosterone predicts leadership within rock hyrax social groups

**DOI:** 10.1038/s41598-023-41958-w

**Published:** 2023-09-08

**Authors:** Yael Goll, Camille Bordes, Yishai A. Weissman, Inbar Shnitzer, Rosanne Beukeboom, Amiyaal Ilany, Lee Koren, Eli Geffen

**Affiliations:** 1 School of Zoology, Tel Aviv University, Tel Aviv, Israel; 2 The Mina and Everard Goodman Faculty of Life Sciences, Bar-Ilan University, 52900 Ramat-Gan, Israel

**Keywords:** Behavioural ecology, Animal behaviour, Social evolution, Social neuroscience, Stress and resilience, Ecology, Neuroscience, Zoology

## Abstract

Group movement leadership is associated with higher risks for those in the front. Leaders are the first to explore new areas and may be exposed to predation. Individual differences in risk-taking behavior may be related to hormonal differences. In challenging circumstances, such as risk-taking leadership that may pose a cost to the leader, cortisol is secreted both to increase the likelihood of survival by restoring homeostasis, and to mediate cooperative behavior. Testosterone too has a well-established role in risk-taking behavior, and the dual-hormone hypothesis posits that the interaction of testosterone and cortisol can predict social behavior. Based on the dual-hormone hypothesis, we investigated here whether the interaction between testosterone and cortisol can predict risk-taking leadership behavior in wild rock hyraxes (*Procavia capensis*). We used proximity loggers, observations, and playback trials to characterize hyrax leaders in three different leadership contexts that varied in their risk levels. In support of the dual-hormone hypothesis, we found that cortisol and testosterone interactions predict leadership that involves risk. Across different circumstances that involved low or high levels of risk, testosterone was positively related to leadership, but only in individuals (both males and females) with low levels of cortisol. We also found an interaction between these hormone levels and age at the low-risk scenarios. We suggest that the close social interactions and affiliative behavior among hyrax females within small egalitarian groups may make female leadership less risky, and therefore less stressful, and allow female leaders to influence group activities.

## Introduction

Testosterone is involved in complex social interactions. Seminal studies on the role of testosterone in male social behavior have suggested that its levels increase in response to a challenge, promoting physical aggression (‘the challenge hypothesis’^1^). Later, ‘the social status hypothesis’^2^ posited that the role of testosterone in pro-social interactions might be as a driver of behaviors that tend to increase an individual’s motivation and ability to acquire and maintain social status. Evolutionary theories imply that risk-taking may have evolved as a behavioral strategy for social status achievement, and that individuals who have much to lose should be more risk-averse than individuals who have little to lose^3^. These individual differences in risk-taking may have a biological basis that involves hormones, such as testosterone and cortisol^4^.

Both the challenge and the social status hypotheses regarding the involvement of testosterone predict an association between testosterone and status-seeking behavior (such as dominance and aggression), but the findings have often been inconsistent^5,6^. The dual-hormone hypothesis (DHH) was developed to explain the inconsistences in research on testosterone and human social behavior^5,6^. This hypothesis posits a framework in which the interaction of testosterone and cortisol can predict status-seeking behavior (e.g., leadership, social status, aggressive and violent behaviors, and risk taking), and therefore suggests an alternative approach to examine risk-taking, whereby testosterone may indirectly impact aggressive and dominant behaviors, and cortisol has a role as a moderator of testosterone. Hence, Mehta and Josephs^6^ suggested that testosterone’s association with status-relevant behavior depends on cortisol levels, such that the positive association between testosterone and status-seeking behavior is stronger when cortisol levels are low, and attenuated when cortisol levels are high (i.e., high and low on a continuum scale hereafter).

Cortisol is released when individuals experience challenging or energetically demanding situations, and it mediates the metabolic and physiological responses resulting from the hypothalamic–pituitary–adrenal (HPA) axis activation. This response system is important in increasing the likelihood of survival when a threat is encountered. The HPA axis, which is generally sensitive to changes in the internal state of the individual, is triggered in response to novel or unpredictable situations^7^, and may also be involved in the behavioral mechanisms that support social group dynamics. Individuals that are consistently challenged by the social hierarchy may be chronically stressed and their HPA axis may be continually activated. Such individuals may be subordinates or dominants, depending on the social system^8^. Although measurements of glucocorticoids have been used to characterize conflicts between dominant and subordinate members of a group^9^, they can also characterize cooperative behavior, and may underlie individual differences in social behavior in wild animals, including affiliation style, parental care^10^, sexual pair bonding and non-sexual bonding^11^.

In this work we examined the association of long-term, integrated testosterone and cortisol levels with leadership in the rock hyrax (*Procavia capensis*), a social mammal that maintains long-term associations in stable social groups^12^. A hyrax group usually comprises of one mature resident male and 3–20 females over 2 years of age with their pups. Like other female egalitarian societies^13^, females in a hyrax group show small differences in social rank, they breed synchronously, rear their pups cooperatively, and live where feeding trees and vegetation patches are abundant. Younger males gradually disperse from their group at the age of 17–24 months or may be forced to disperse by the resident male^14,15^. Bachelors live mostly solitarily on the periphery of the colonies, do not participate in coordinated group activities (Fig. S1), and their interaction with the mixed-sex groups is mainly for mating^15^. Hyrax group members share sleeping dens and travel together to feeding locations and back to the sleeping dens. Hyraxes move in a sequential manner, in which each group member independently advances short distances before joining others in fixed stops. The first animals arrive individually at the final destination and may be alone for up to a few minutes until the next individual arrives, and consequently be exposed to potential predation. Moreover, the predictability of feeding locations makes these sites more dangerous. The Arabian leopard (*Panthera pardus nimr*), now extinct in Ein Gedi (since the early 2010s), used to ambush hyraxes near their sleeping dens or in feeding bushes^16^. Today, gray wolves (*Canis lupus*) and red foxes (*Vulpes vulpes*) are the common predators of hyraxes. We therefore suggested that the hyraxes' riskiest move is that of their arrival at a feeding location^17^. Hyraxes travel short distances that are within sight, but such travel may require experience to decide where to forage and minimize the risk of predation.

Social animals coordinate their activities with other members of the group to stay together and maintain the benefits of group living. Some individuals might exert greater influence than others on group activity, depending on their specific traits, and these individuals are often defined as leaders^18^. We have recently defined the first animal to arrive as the leader, as this individual influences the movement of others, and therefore we classify hyrax leadership as a behavior that involves risk^17^. Hyrax males and females fulfil different roles within their egalitarian social group, and we found that they take on different leadership positions, depending on the context: younger resident males with higher levels of cortisol and lower levels of testosterone, and females with lower levels of testosterone, were more likely to lead in everyday coordinated activities such as coordinated movement towards feeding locations^17^. In Goll et al.^17^ we identified leadership that involves risk in rock hyraxes and characterized hyrax leaders in different contexts. Here we further investigated leadership behavior, and examined whether the dual-hormone hypothesis can predict leadership that involves risk in rock hyraxes. We evaluated the plausibility of the DHH by testing for the presence of an interaction between testosterone and cortisol, which was not tested in the models presented in Goll et al.^17^.

We used the dual-hormone hypothesis as a framework for this study and for setting up several predictions to be tested. Because the DHH considers cortisol as a potential moderator of testosterone’s association with behavior that involves risk, we therefore measured the testosterone and cortisol interaction in three leadership contexts that involve risk (low risk: movement towards feeding trees and morning emergence from sleeping dens, high risk: predator defense events; see “Methods” section for further details). Following the DHH predictions, we hypothesized that cortisol and testosterone would interact to predict behaviors related to leadership that involves risk, depending on the context. Testosterone in the rock hyrax is associated with social status, such that the more dominant males have higher testosterone levels, and the more dominant females have lower testosterone levels^19^. These males and females also demonstrate the highest copulation success^20^. Although we hypothesized that these males and females would attract other group members to follow them when they lead, we found that both females and males with low testosterone were more likely to lead. Also, although front position leadership involves a potential predation risk, only male leaders showed high levels of cortisol^17^. While the stress response helps to mediate adaptation to short-term physical stressors, it is pathogenic when secreted chronically. If animals are consistently socially stressed by the dominance hierarchy, they exhibit hyperactivity of the HPA axis and undergo neurobiological changes^8^. However, the stress response also motivates animals to act in a way that gets them what they need, and it includes engaging socially. In several bird species, for example, an increased threat of predation promotes cooperation^21^. Hyrax group females are likely to help the leader (e.g., act as sentinel or mob a predator), and we therefore suggest that the close social associations among hyrax females make female leadership less risky, and therefore less stressful.

## Results

The data in this study was collected during 2015–2018 from five social groups. Our data is composed of three datasets of observations of leadership behavior that are associated with low and high risk scenarios (see details in the “Methods” section). We examined the overall relationship between hair testosterone and cortisol levels in 54 females and 33 males, sampled over multiple years using a random effect mixed model. There was no significant difference in cortisol (F_1,81_ = 0.12, *P* = 0.728) or testosterone (F_1,50_ = 0.07, *P* = 0.790) levels between the sexes. We did detect a significantly positive association between testosterone and cortisol levels in both males (r^2^ = 0.71, model estimate = 0.89, F_1,35_ = 20.3, *P* < 0.0001) and females (r^2^ = 0.31, model estimate = 0.47, F_1,74_ = 24.4, *P* < 0.0001). Overall, animals with high testosterone levels also had high cortisol levels.

**Figure 1:**
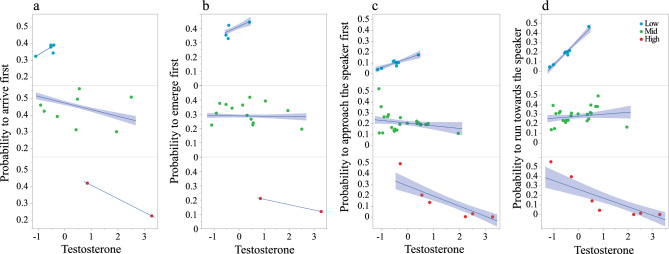
Interaction plots for the probability to lead as a function of standardized testosterone level for low (Lower than mean − SD, blue), mid (mean ± SD, green), and high (higher than mean + SD, red) standardized cortisol levels (i.e., see legend). The interaction plots showing the probability of a female to arrive first at a base station (**a**; low risk), the probability of a female to emerge in the morning from a den (**b**; low risk), the probability of an individual to approach first the speaker in a pup-scream trial (**c**; high risk), and the probability of an individual to run towards a speaker in a pup-scream trial (**d**; high risk). Linear trend lines and 95% CI are presented only for illustration.

The most notable result observed in nearly all the models is the strong evidence for interaction between cortisol and testosterone levels (Tables 1, 2 and 3). The evidence for interaction was strong (i.e., *P* ≤ 0.044) in all models we tested but in two: coordinated movement towards a feeding site by females (*P* = 0.417, Table 2A and *P* = 0.065 for the extended model in Table 3A). Furthermore, the model coefficient in all the significant cortisol–testosterone interactions was negative, suggesting a similar trend. Examination of this interaction across models revealed that the likelihood of leading (both males and females) with low cortisol increased with the rise in testosterone but decreased in animals possessing high cortisol levels (Fig. 1, Figs. S3–S4). The cortisol and testosterone interactions were prominent in both low- and high-risk scenarios (i.e., low-risk coordinated movement and morning emergence sequences and high-risk predator defense events).

**Table 1:** Coordinated movement sequences; low risk.

Term	Estimate ± (SE)	Wald $\upchi _{1}^{2}$	*P*	Total effect
Both sexes n = 518, 19 individuals				
Body weight	− 0.29 (0.21)	1.8	0.178	0.71
Cortisol	1.90 (1.06)	3.2	0.071	0.59
Testosterone	− 0.85 (1.33)	0.4	0.523	0.31
Body weight * Cortisol	− 0.67 (0.44)	2.4	0.123	
Body weight * Testosterone	0.34 (0.52)	0.4	0.516	
Cortisol * Testosterone	− 0.17 (0.06)	7.9	**0.005**	
Females n = 351, 15 females				
Body weight	0.69 (0.15)	22.6	** >0.001**	0.54
Cortisol	− 1.74 (0.92)	3.6	0.057	0.32
Testosterone	− 2.12 (0.82)	6.7	**0.010**	0.63
Body weight * Cortisol	0.85 (0.39)	4.8	**0.029**	
Body weight * Testosterone	0.86 (0.34)	6.3	**0.012**	
Cortisol * Testosterone	− 0.25 (0.02)	163.7	** >0.001**	

The effect of body weight, hair cortisol and hair testosterone on the order of arrival of hyraxes to a base station for the pooled data of both sexes, and for females. Data on the order of arrival was collected by proximity loggers and analyzed using mixed ordinal logistic GEE model. VIF ≤ 4.3 for all predictors. Significant effects and interactions are indicated in bold.

**Table 2:** Coordinated movement and morning emergence sequences; low risk.

Term	Estimate ± (SE)	Wald $\upchi _{1}^{2}$	*P*	Total effect
A. Order of movement towards a feeding site				
Males n = 73, 6 males				
Body weight	− 0.14 (0.12)	1.3	0.252	0.57
Cortisol	− 1.80 (2.95)	0.4	0.541	0.41
Testosterone	− 15.94 (1.38)	133.6	**< 0.001**	0.55
Body weight * Cortisol	0.33 (0.97)	0.1	0.733	
Body weight * Testosterone	5.80 (0.48)	149.0	**< 0.001**	
Cortisol * Testosterone	− 0.33 (0.16)	4.1	**0.044**	
Females n = 177, 15 females				
Body weight	0.57 (0.74)	0.6	0.439	0.94
Cortisol	− 10.62 (2.18)	23.8	**< 0.001**	0.44
Testosterone	2.88 (1.81)	2.5	0.112	0.20
Body weight * Cortisol	4.52 (0.97)	21.9	**< 0.001**	
Body weight * Testosterone	− 1.49 (0.78)	3.7	0.055	
Cortisol * Testosterone	0.08 (0.10)	0.7	0.417	
B. Order of emergence from a sleeping den				
Both sexes n = 315, 21 individuals				
Body weight	0.34 (0.37)	0.8	0.363	0.77
Cortisol	− 1.90 (1.71)	1.2	0.266	0.22
Testosterone	0.59 (0.98)	0.4	0.544	0.25
Body weight * Cortisol	0.72 (0.74)	0.9	0.333	
Body weight * Testosterone	− 0.20 (0.43)	0.2	0.643	
Cortisol * Testosterone	− 0.19 (0.09)	4.3	**0.037**	
Females n = 233, 15 females				
Body weight	− 0.87 (0.29)	8.9	**0.003**	0.86
Cortisol	− 0.24 (1.06)	0.0	0.824	0.13
Testosterone	0.19 (0.79)	0.1	0.808	0.40
Body weight * Cortisol	0.04 (0.42)	0.0	0.932	
Body weight * Testosterone	− 0.02 (0.32)	0.0	0.962	
Cortisol * Testosterone	− 0.16 (0.03)	29.2	**< 0.001**	

The effect of body weight, hair cortisol and hair testosterone on the order of movement towards feeding sites A and on the order of emergence from sleeping dens B. Data for both A and B were obtained by direct observations and analyzed using mixed ordinal logistic GEE model. VIF ≤ 2.4 for all predictors. Significant effects and interactions are indicated in bold.

**Table 3:** Predator defense events; high risk.

Term	Estimate ± (SE)	Wald $\upchi _{1}^{2}$	*P*	Total effect
A. First to reach the speaker				
Both sexes n = 85, 25 individuals				
Extended model				
Body weight	1.62 (1.33)	1.5	0.223	0.44
Cortisol	− 2.90 (4.33)	0.4	0.503	0.51
Testosterone	− 0.82 (3.84)	0.0	0.831	0.70
Body weight * Cortisol	1.24 (1.73)	0.5	0.475	
Body weight * Testosterone	0.23 (1.44)	0.0	0.876	
Cortisol * Testosterone	− 0.91 (0.49)	3.4	0.065	
Restricted model				
Cortisol	0.46 (0.39)	1.4	0.236	0.70
Testosterone	− 0.02 (0.34)	0.0	0.956	0.97
Cortisol * Testosterone	− 0.75 (0.35)	4.6	**0.032**	
Females n = 69, 22 females				
Restricted model				
Cortisol	0.02 (0.52)	0.0	0.967	0.83
Testosterone	− 0.11 (0.54)	0.4	0.844	0.77
Cortisol * Testosterone	− 1.12 (0.55)	4.1	**0.042**	
B. Running towards the speaker				
Both sexes, n = 85, 25 individuals				
Extended model				
Body weight	1.63 (1.16)	2.0	0.159	0.18
Cortisol	− 2.12 (3.81)	0.3	0.578	0.86
Testosterone	− 1.96 (3.63)	0.3	0.589	0.45
Body weight * Cortisol	0.62 (1.53)	0.2	0.686	
Body weight * Testosterone	0.87 (1.38)	0.4	0.526	
Cortisol * Testosterone	− 1.61 (0.54)	9.0	**0.003**	
Restricted model				
Cortisol	− 0.10 (0.43)	0.1	0.812	0.95
Testosterone	0.37 (0.41)	0.8	0.365	0.67
Cortisol * Testosterone	− 1.22 (0.44)	7.5	**0.006**	
Females n = 69, 22 females				
Restricted model				
Cortisol	− 0.68 (0.64)	1.1	0.290	0.83
Testosterone	0.51 (0.53)	1.0	0.339	0.77
Cortisol * Testosterone	− 1.86 (0.58)	10.4	**0.001**	

The effect of body weight, hair cortisol and hair testosterone on the probability to first reach the speaker A and on the probability of running towards the speaker B in both sexes and in females. Data on both order of movements were obtained from pup screams trials and analyzed using mixed nominal logistic GEE model. VIF ≤ 3.1 for all predictors. Significant effects and interactions are indicated in bold.

Body weight, used in these datasets, is a reliable index of age in rock hyraxes (r^2^ = 0.94 and 0.95 for females and males, respectively^15^). Body weight was directly associated with the probability of leading only in females in the emerging from sleeping dens scenario (Table 2B), with heavier females more likely to lead. However, we detected a significant effect of body weight through its interactions with cortisol and testosterone (Tables 1 and 2, Figs. S3–S4). These significant body weight interactions had positive model coefficients, revealing that small (i.e., light) individuals tended to display a lower probability of leading with an increase in cortisol and testosterone. In contrast, heavy individuals tended to keep stable or increase the probability to lead with an increase in cortisol and testosterone. Interactions between body weight and cortisol or testosterone were only detected in the low-risk scenarios.

We note that in the pup-scream playbacks (predator defense events, high risk), the distance of the animal to the speaker was not a significant predictor of the probability of reaching the speaker first (GEE: χ^2^_1_ = 0.05, *P* = 0.976) or the probability of running towards the speaker (GEE: χ^2^_1_ = 1.83, *P* = 0.401). Therefore, we did not include the distance to the speaker as a predictor in the models presented in Table 3.

Last, contour plots of the probability of leading as a function of cortisol and testosterone enabled us to evaluate the hormonal profile of a typical leader under the different risk scenarios (Figs. S5–S7, summarized in Table 4). The coordinated movement data from the proximity loggers (low risk) showed that the most likely individuals (i.e., both males and females) to reach a base-station first were those with low levels of testosterone and mid-levels of cortisol (Fig. S5a), as well as females with mid-levels of both testosterone and cortisol and the largest body weight (Fig. S5b–d). The coordinated movement data from the direct observations of behavior (low risk) revealed that females with lower cortisol and testosterone levels demonstrated the highest likelihood of emerging first from a sleeping den (Fig. S6b). The probability of reaching a feeding site first was highest for males with small body weight and low levels of testosterone and cortisol (Fig. S6c,d). In contrast, females that showed the highest likelihood of reaching a feeding site were both small females with low cortisol levels and large females with high cortisol levels (Fig. S6f), as well as those with low testosterone levels (Fig. S6e). The pup-scream playback data, which reflected high-risk scenarios, revealed that the females possessing the lowest testosterone levels and mid-range cortisol levels were the most likely to reach the speaker first (Fig. S7a). The females that were most likely to run towards the speaker after a playback were those that had mid-levels of testosterone and low levels of cortisol (Fig. S7b).

**Table 4:** Summary of hypothesized and observed hair testosterone and cortisol levels in leaders across the different contexts.

Data type	Context Risk level	Sex	Hypothesized testosterone level	Hypothesized cortisol level	Observed testosterone level	Observed cortisol level
Proximity loggers	Coordinated movement towards feeding trees low	Both sexes	Mid	Mid	Low	Mid
		Females	Low	Mid	Mid	Mid
Direct observations	Coordinated movement low	Males	High	High	Low	Low
	Morning emergence low	Both sexes	Mid	Mid	Low	Low
		Females	Low	Mid	Low	Low
Pup screams playback	Predator defense high	Females	Low	High	Low	Mid

We refer in this table to three steroid levels on the continuum scale. See methods for definitions.

## Discussion

We found that the interaction between long-term measurements of hair cortisol and testosterone, which are related to social status, copulation success and social state in rock hyraxes^15,19,20^, can predict leadership behavior. Across all three contexts of low and of high risk, testosterone was positively related to leadership in both males and females, but only in individuals with low cortisol levels. Thus, our current findings support the DHH, and its predictions for testosterone and cortisol interaction in both male and female hyraxes. However, we cannot attribute causality to those associations. Leadership may contribute to testosterone and cortisol interaction, or testosterone and cortisol interactions may lead to leadership behavior. In wild meerkats (*Suricata suricatta*), for example, variations in glucocorticoids can influence cooperative behavior (alloparental behavior^22^), and cooperative behavior can also modulate hormone levels and lead to elevated glucocorticoids levels^23^.

The original dual-hormone hypothesis suggested that high testosterone levels predicted higher social status only when cortisol levels were low^6^. That hypothesis predicted an interaction between levels of testosterone and cortisol in risk-taking; and, in men, risk-taking was indeed shown to be associated with high levels of testosterone and low levels of cortisol^24^. Generally, hyraxes with high levels of hair cortisol also showed high levels of hair testosterone. Hyrax leaders, however, had low levels of cortisol and high levels of testosterone, and this interaction of the two hormones was similar to the original interaction suggested by Mehta & Josephs^6^. The broader perspective of this theory suggests that, generally, the statistical interaction of testosterone and cortisol predicts status-related behaviors. For example, in women, an inverse association between testosterone and status was found for those who have relatively high cortisol levels^25^.

In Goll et al.^17^ we identified leadership that involves risk in rock hyraxes, defined hyrax leaders as those individuals whose influence on the group is greater than that of others, and demonstrated that this leadership is not related to lactation or hunger. We found that leadership in hyraxes is characterized by specific traits for male leaders: younger resident males had high levels of cortisol and low levels of testosterone. In contrast, each one of the group females may lead coordinated activities at different times^17^. Here we specifically tested the DHH, and have demonstrated that in hyraxes this theory can predict leadership that involves risk. We found that the interaction between testosterone and cortisol can predict leadership behavior: leaders are more likely to lead if they had high testosterone levels, but only if their cortisol levels were low. We suggest that the variation that we found in cortisol and testosterone interaction in females across contexts might stem from the nature of the long-term social interactions among female hyraxes in their small, relatively stable, egalitarian groups^26,27^. Female group members may act as sentinels and provide their leaders with accurate information regarding risks in the environment. They are also likely to help the leader mob a predator (YG, pers. obs.). Consequently, the close social associations among hyrax females might make leadership less risky for them, and therefore less stressful, and allow for more female leaders to influence group activities. In numerous species, both females and males occupy leadership positions to some or to equal extent, and in a small fraction of them, including killer whales (*Orcinus orca*)^28^, African lions (*Panthera leo*)^29^, spotted hyenas (*Crocuta crocuta*)^30^, bonobos (*Pan paniscus*)^31^, ring-tailed lemurs (*Lemur catta*)^32^, and African bush elephants (*Loxodonta africana*)^33^, females emerge as strong leaders during collective behaviors across multiple contexts.

In addition, we found a strong interaction between long-term integrated testosterone and cortisol levels and body weight, which is a reliable index of age in rock hyraxes^15^. Glucocorticoids mediate age-dependent transitions through the life-history stages, by altering the physiology and behaviors that influence life-history traits such as age-specific growth and reproduction, as well as survival^34^. Our findings suggest that these two hormones may influence leaders differently at different stages of their life. However, we cannot attribute causality to these associations, and therefore cannot determine whether leadership influences hormonal levels, or whether hormonal levels influence leadership.

In this study, testosterone and cortisol were quantified in hyrax hair, a measurement that reflects long-term trends rather than acute levels. Hair was sampled at the beginning of each field season (March–April), and therefore represents the integrated hormone levels of the 1–2 months prior to the breeding and mating seasons, and the observed leadership behavior (shaved hair regrows within 1–2 months). Circulating steroids are assumed to be embedded in the hair, enabling quantification of integrated steroid levels over the period of hair growth. These levels are unaffected by diurnal changes or by the momentary stress of capture^35^. We expected the hair samples to represent baseline measurements of hormone levels, and therefore suitable to be correlated with behaviors that are displayed over a long period of time such as leadership. In stressful situations like pup predation, however, circulating hormone levels may be elevated, and the long-term trends we found do not reflect short-term testosterone and cortisol interactions. In addition, consistent with prior findings^36^, no sex differences in hair testosterone levels were found, and we therefore expected to find an interaction between testosterone and cortisol in both male and female hyraxes. However, characterizing the hormonal profile of male leaders in this study was not possible in many of the contexts because hyrax groups comprise several adult females but often only a single mature resident male, and adult bachelor males do not participate in coordinated group activities (Fig. S1). In most cases, therefore, we were unable to examine male leaders’ testosterone and cortisol interaction in leadership that involves risk.

## Conclusions

The endocrine system and release of hormones have a vast and complex influence on social behavior. The DHH focuses on interactions between cortisol and testosterone as predictors of status seeking behavior like leadership (i.e., individual’s influence on collective group behavior^37^). Here we found that testosterone and cortisol interaction predicted leadership status that involves risk-taking in rock hyraxes, a finding that provides strong support for the DHH. We found that in females, high levels of cortisol did not accompany the risk involved in maintaining a leadership position in coordinated activities that involve risk. This result possibly reflects the important impact of female social interaction and support within a group. It is possible that the relationship between testosterone and cortisol may have evolved flexibly to buffer the potentially adverse effects of stress on health, and ultimately on fitness.

## Methods

### Ethical statement

The study was conducted under annual permits from the Israeli Nature and Parks Authority (NPA) for capturing, handling and tagging the hyraxes at the Ein Gedi Nature Reserve (2015/40768, 2016/41174, 2017/41507, 2018/41880). All procedures performed in this study involving animals were in accordance with the ethical standards of the NPA and the state of Israel, and the ARRIVE guidelines.

### Field protocol

This research is part of our long-term study on hyrax sociality, conducted since 1999 at the Ein Gedi Nature Reserve, Israel (31° 28′ N, 35° 24′ E). Our field protocol includes trapping, handling, and observation procedures in the field, and follows our previously published protocols^15, 36^. All animals in this study were trapped for tagging and sampling. We used live box traps (Tomahawk Live Trap Co, Tomahawk, WI) baited with cabbage and kohlrabi. The traps were set at dawn at shady sites and inspected after 3–4 h. Trapped animals were anesthetized by intra-muscular injection of ketamine hydrochloride (10 mg/kg). Hyraxes were individually marked using subcutaneous transponders (DataMars SA), earrings and numbered collars (collar weight 5 g; range of 0.125–0.2% of hyrax body weight), and were weighed and measured. During 2017–2018 adult hyrax members of the group were fitted with a proximity logger (E2C 162A; SIRTRACK, New Zealand), attached to a collar (weight ca. 30 g, less than 1% of body weight). All treatments were performed in the shade to avoid overheating. Following anesthesia recovery (at least 120 min), the animals were released back at their capture sites and resumed full normal activity, and the traps were secured open until the next trapping session. Over the course of the study, the hyraxes were observed daily, and no adverse effects of the loggers were observed. Proximity base-stations (E2B176A Sirtrack) were deployed in central shared locations (e.g., shared feeding areas, sleeping dens). The E2C-171-A Sirtrack proximity loggers detect and identify each other when in a radius of up to 2 m^38^ via individual UHF transceivers. The base stations recorded the order of arrival of all collared individuals at these areas (including time and date of arrival, time intervals of 1 s between two individuals) within a range of up to ~ 6 m from a base station. At the end of the field season (4–5 months after the loggers were on) the hyraxes were trapped again to recover the loggers, which were replaced with numbered collars.

### Datasets

We collected data by means of direct observations, proximity loggers and playback trials, which were partially used in Goll et al.^17^. These datasets were all collected from the same study site but using different techniques, often from different individuals and on different time scales, thus providing three separate and non-comparable datasets. Based on our long-term study, which spans more than 20 years of observations, we classified contexts as high risk those in which predation had been observed frequently (i.e., pup predation), and low risk those in which predation had been observed rarely (i.e., predator ambush at a feeding tree). We had used parts of these datasets previously to characterize male and female hyrax leadership in a different context, comprising: (a) coordinated movement; (b) morning emergence order; and (c) predator defense^17^.

The first dataset, composed of coordinated movement sequences (low risk, a), was collected during 2017–2018 and comprised the sequences of the arrival of collared group members at base-stations located at feeding trees and sleeping sites (> 40 m from one another) in the Arugot and David gorges (n = 881 leader/follower visits). Two groups (Isiim and Cube) visited base-stations located in six (in 2017) and three (in 2018) different locations in Arugot, and two groups (Window and Hill) visited base-stations located in three different locations (in 2017 and 2018) in David. Leaders/followers visits were detected during 50 ± 21 days in each location.

The second dataset, composed of coordinated movement and morning emergence sequences (low risk, a,b), was collected during 2017–2018 and comprised observations on the order of emergence from sleeping dens in the morning (n = 472 observations) and the order of movement towards a feeding tree (n = 400 observations) in the Arugot and David gorges. Three groups were observed in Arugot (Isiim, Cube, and Sukkot) for 123 ± 30 days, and two groups were observed in David (Hill and Window) for 106 ± 42 days.

The last dataset, composed of predator defense events (high risk, c), was compiled from the pup-scream playback trials that were conducted in the Arugot and David gorges during 2015–2018, and comprised 147 observations on the order of individuals approaching the speaker following the playback. Three groups were observed in Arugot (Isiim, Cube, and Entrance) and two groups were observed in David (Hill and Window).

### Base-station arrival sequences (coordinated movement, low risk)

For the base-station data using proximity loggers, we defined coordinated movement events as those in which one or more group members were tracked following a leader; and in which the time window between two following individuals did not exceed the upper 95% CI value of the time window in all base-station events (< 18 min). Only for those events that included followers, was the first individual tracked arriving at a base-station defined as a leader, as in Goll et al.^17^. If time interval between two individuals was less than 2 s, both individuals defined as arrived synchronously.

### Coordinated movement and morning emergence sequences (low risk)

All hyrax group members regularly share sleeping dens and travel together in sequential movement pattern to feeding locations or back to sleeping dens, while exposed to potential predation. The predictable location of their feeding sites makes such sites more susceptible to the presence of predators, and so the riskiest move for hyraxes displaying tandem movement is that of their arrival at the feeding location^39, 40^. We therefore defined the first animal to arrive at the destination as the leader, but only when followed by at least one other group member^17^. To identify those individuals with a stronger influence on the group, we defined a minimum time window of ≤ 15 min (based on our empirical data) for group members to follow one another. Only leader–follower sequences during which one or more group members were observed following one another within this defined time window were defined as ‘coordinated movement events’, and were included in the analysis^17^. Sequences of group members moving together but with a greater time interval between them were removed from the analysis. Similarly, in the emergence context, the first adult animal to emerge from the sleeping den in the morning, within the defined time window, was defined as the leader. For both contexts, analysis was conducted for adult group members only (> 2.5 years), as social cohesion is more crucial for juveniles than for adults, and juveniles consequently have much less influence on group movement decisions^41^. We defined ‘coordinated movement events’, or ‘morning emergence events’, as those in which one or more group members were observed following a leader within a defined time window (< 15 min). Only in those events that included followers, was the first individual arriving at the destination, or emerging from the sleeping den, defined as a leader as in Goll et al.^17^. Moreover, we defined leadership in this context as the probability of being first to arrive, or emerge, or approach in various contexts during a period of 2–4 months (for example, for a period of 3 months, a particular individual is more likely to be the first to arrive at a feeding tree, and is therefore defined as a leader).

### Playback trials (predator defense events, high risk)

Pup-scream playbacks in the field were conducted to simulate predation scenarios. We followed the protocol of Ilany et al.^42^, which had demonstrated that group females strongly reacted to the playback of pup screams (with a similar response to screams from their own group pups and those from out-group pups) but were non-responsive to male songs (i.e., control). In each trial session, a remote-controlled speaker (FoxPro Scorpion X1B speaker, FOXPRO Inc., Lewistown, PA, U.S.A.) was placed before dawn in a location at which hyraxes frequently arrive. When a hyrax group was observed within 10–30 m from the speaker, a pup-scream playback was played via a FoxPro speaker using a TX200 remote control (volume was similar to natural pup-scream sounds; Ilany et al.^42^), and all hyrax activities were recorded. The order in which the hyraxes approached the speaker was recorded from first to last, with the first individual to approach (~ 40 cm from the speaker) defined as the leader^17^. The distances of the hyraxes from the speaker before and during playback were estimated from direct observations and assigned to three categories: near (< 20 m), far (> 20 m), and not seen before playback. The pup screams used in the trials had been recorded by Ilany et al.^42^ during the marking and measuring of captured pups. To minimize pseudo-replications, we used nine independent (i.e., from different individuals) recordings of pup screams. As control playbacks, we used recordings of male songs (n = 9 different males), which had been previously used by Ilany et al.^42^. No more than one experiment was performed in the same week on each group, to prevent habituation to the treatment.

### Hormonal assay

Levels of hair cortisol and testosterone were measured following previously published protocols^15,19,36^. Hair-testing provides a long-term profile of integrated steroid levels^43^. Hair samples were collected once a year by cutting from the thigh of all trapped hyraxes. We analyzed a total of 136 hair samples from 87 individuals that had been collected between March and April 2015–2018. For each individual one sample per year was analyzed, and levels of hair cortisol and testosterone from that year were correlated with leadership characteristics for that specific year's field season (e.g., cortisol and testosterone levels from 2015 were associated only with leadership characteristics from the 2015 field season). If any individual lacked hair sample from a specific year, its leadership data from that year was omitted.

Samples were washed twice in isopropyl alcohol (Sigma-Aldrich Israel LTD, Rehovot, Israel) while gently shaken (100 rpm; 3 min). Hair samples were dried, and 20 mg or 30 mg (for testosterone and cortisol, respectively) were weighed to the nearest 0.01 mg by electronic balance (BJ610c, Precisa, Dietikon, Switzerland), sonicated with methanol (4 ml, 30 min; Sigma-Aldrich Israel LTD, Rehovot, Israel) and then incubated with shaking (50 °C, 130 rpm, 20 h). The methanol was evaporated with gaseous nitrogen (45 °C), and the samples were run in duplicates on a commercial enzyme-linked immunosorbent assay (ELISA) kit for testosterone and cortisol quantification (Testosterone: EIA-1559, DRG International Inc., Springfield Township, NJ, U.S.A.; Cortisol: Kit no. 1-3002, Salimetrics, Ann Arbor, MI, USA) following the kit protocol, after restoration with the zero-standard provided. Samples were run in duplicates. The testosterone kit had been previously validated for hyrax hair^15,19,36^. Duplicates of the pool quantified on different days gave an inter-assay coefficient of variation of 6.16% and running six duplicates of a pool of hyrax hair on the same plate gave an intra-assay variability of 4.69%^20^. For the cortisol kit, the intra-assay variability for six duplicates of the hyrax hair pool run on a single plate was 1.26%. Duplicates of the pool quantified on different days gave an inter-assay coefficient of variation of 6.52%. Serial dilutions of the pool showed parallelism with the provided cortisol kit standard (standard: r^2^ = 0.96, t_(4)_ = 9.8, *P* = 0.0006; samples: r^2^ = 0.97, t_(6)_ = 14.6, *P* < 0.0001). Slopes of the standard and samples were non-significantly different (F_(1,10)_ = 3.1, *P* = 0.108). Linearity was demonstrated between 5 and 60 mg of hair. Addition of a known amount of cortisol to the pool gave a recovery of 101.2%. Cross-reactivity between the testosterone antibody and cortisol was reported to be < 0.1%, and between the cortisol antibody and testosterone 0.006%. Last, to control for the variance in testosterone and cortisol levels associated with processing at different times, we standardized levels by analysis session.

### Statistical analyses

We analysed three different independent datasets, as detailed in Goll et al.^17^. Briefly, the first dataset was collected from the base-stations and comprised sequences of arrival of group members at the base-stations (Table 1). The second dataset was compiled from observations on the order of emergence from sleeping dens in the morning and the order of movement towards a feeding site (Table 2). The third dataset was compiled from the pup-scream trials (Table 3). The small sample size of males (only four resident males were fitted with a proximity logger, and only three resident males participated in the pup-scream playback trials) prevented us from performing an independent analysis for males (i.e., our model for males did not converge). Therefore, we analyzed for both sexes together (whenever possible), as well as separately for females.

We used logistic mixed models to test for the effect of cortisol, testosterone and body weight on the probability of being a leader. We utilized Generalized Estimating Equations (GEE) and logistic response as our mixed model framework as implemented in SPSS (version 26, IBM Inc). We separately analyzed the three different independent datasets, and performed the analysis separately for males and females whenever possible, because hyrax male and female leaders are characterized by different traits^17^. For the base-stations and observations datasets we used a mixed ordinal logistic model, and for the playback trials dataset we used a mixed nominal logistic model. The dependent variables in the mixed ordinal logistic model were the order of arrival or emergence of individuals (Tables 1 and 2), while in the mixed nominal logistic model the dependent variables were binary (e.g., running towards the speaker or not; Table 3). In models where both body weight and its interactions were not significant, we also tested a restricted model with only testosterone and cortisol as predictors. Individual identity was set as a random effect in all models. We did not include group identity as an additional random effect since adding it did not change the model results. For effect size, we used the total effect, a randomization approach that is independent of model type and fitting method^44^. The total effect combines the relative contribution of each predictor both alone and through the interactions with the other predictors. Variable inflation factor (VIF) lower than 10 indicated non-collinearity between predictors.

Last, we referred to the continuous scale of steroid levels as three categories (low, medium, and high). We used non-parametric tolerance intervals (i.e., confidence interval for a specified proportion of the population) for 75% of the population at 1 − α = 0.95 to divide between these levels. Standardized testosterone values below − 1 were considered low and those above 1 were considered high. Standardized cortisol values below − 1.1 were considered low and those above 1.5 were considered high (Fig. S2).

### Supplementary Information

Supplementary Figures.

Supplementary Table S1.

## Data Availability

Data is available as a supplementary Excel table (Table S1).
