# Supplementary material for: The interaction between cortisol and testosterone predicts leadership within rock hyrax social groups
Source: Sci Rep. 2023 Sep 8;13:14857. doi: 10.1038/s41598-023-41958-w (PMC10491601; doi:10.1038/s41598-023-41958-w)
Supplement: Supplementary file 1 — Supplementary Figures. [file 41598_2023_41958_MOESM1_ESM.pdf]

The interaction between cortisol and testosterone predicts leadership within rock hyrax social groups

Yael Goll[a], Camille Bordes[b], Yishai A. Weissman[b], Inbar Shnitzer[b], Rosanne Beukeboom[b], Amiyaal Ilany[b], Lee Koren[b§], Eli Geffen[a§]

[a] *School of Zoology, Tel Aviv University, Tel Aviv, Israel*

[b] *The Mina and Everard Goodman Faculty of Life Sciences, Bar-Ilan University, Ramat-Gan, 52900, Israel*

[§] Equal contribution

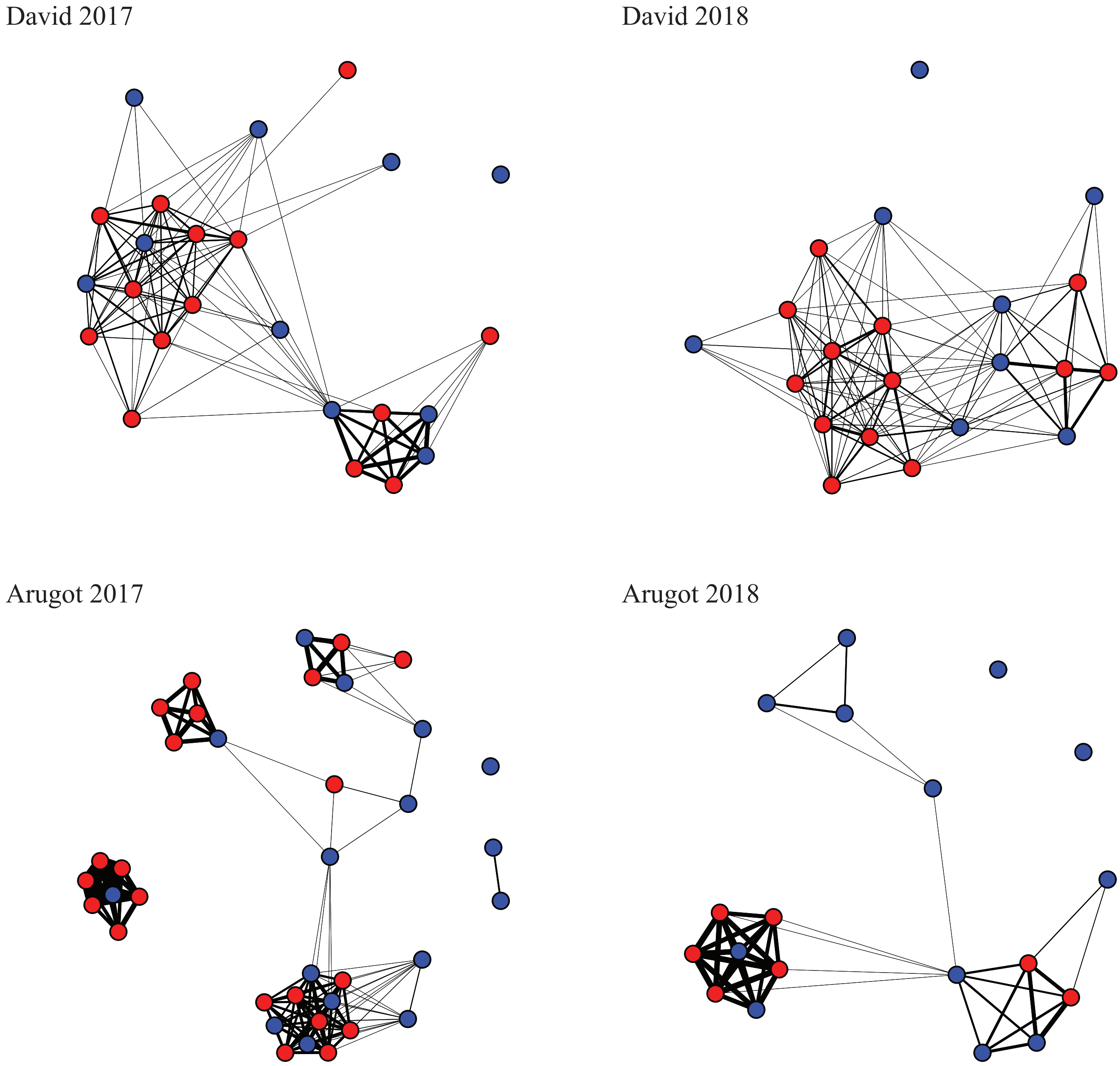

Fig. S1: Social networks in David and Arugot creeks during 2017-2018. Blue and red nodes denote males and females, respectively. The width of ties denotes association strength.

a

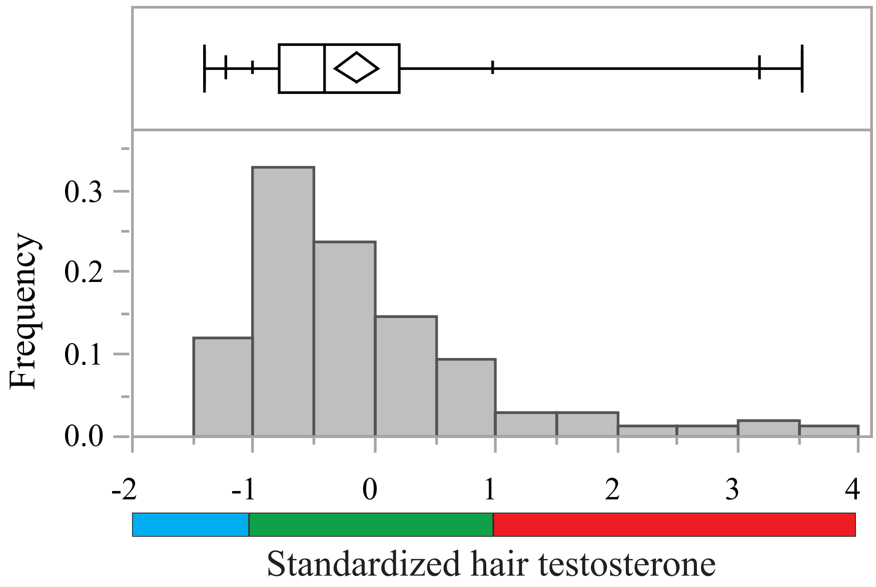

b

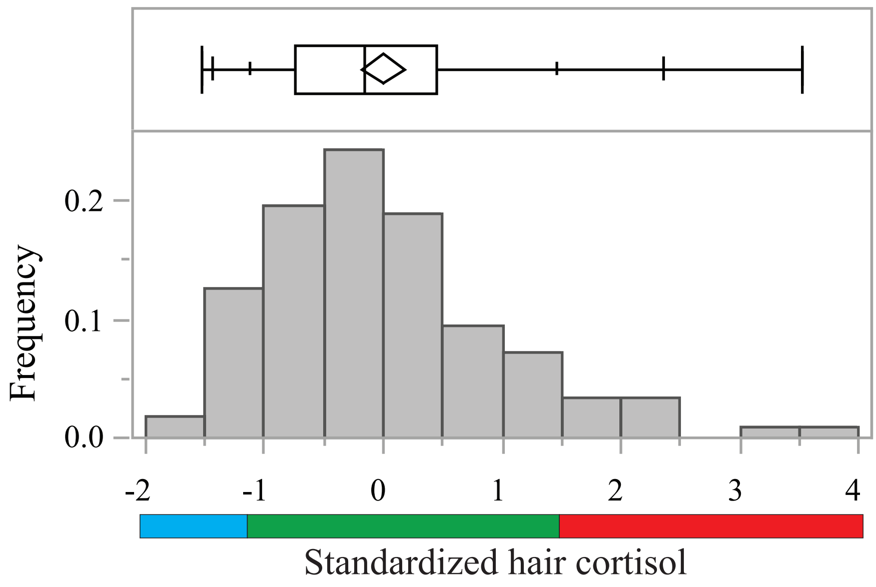

Fig. S2: Distribution of standardized hair testosterone (a; n=120) and cortisol (b; n=129) levels in rock hyraxes. Above the bars, a quantile box plot showing the quantiles for the minimum, 2.5%, 10%, 25%, 50%, 75%, 90%, 97.5%, and the maximum. Blue, green, and red bars denote the range of low, mid, and high levels of testosterone and cortisol, respectively.

a

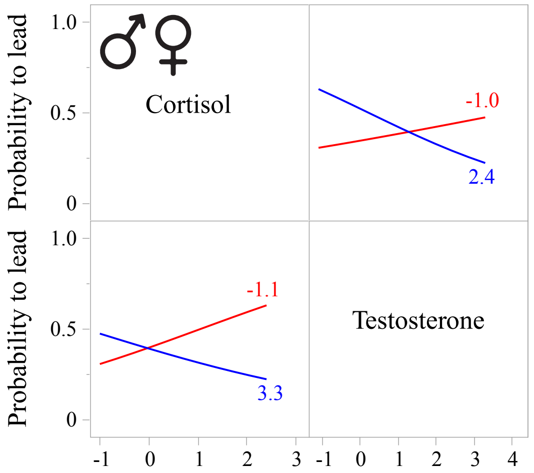

b

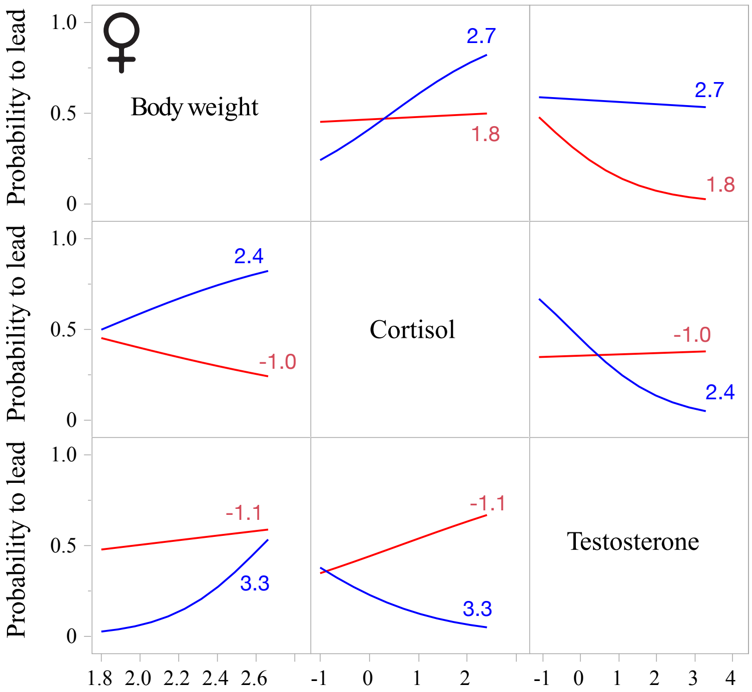

Fig. S3: Interaction profiles showing the effects body weight, cortisol and testosterone levels on the probability to lead in both males and females combined (a; Table 1) and females only (b; Table 1). Blue curves represent maximal values and red curves minimal values of the predicor labelled in the row. Data on the order of arrival was collected by proximity loggers and analyzed using mixed ordinal logistic models (GEE).

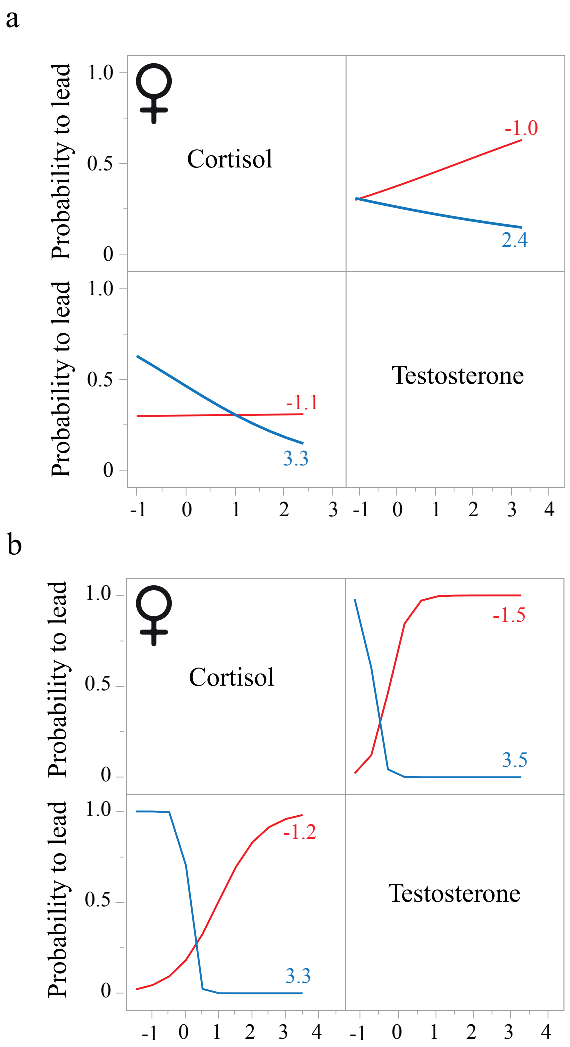

Fig. S4: Interaction profiles showing the effects cortisol and testosterone levels on the probability to lead in the order of emergence (a; Table 2) and running towards the speaker (b; Table 3). Blue curves represent maximal values and red curves minimal values of the predicor labelled in the row. For example, for the highest cortisol level (i.e. 3.5), higher testosteron level is reducing the probability to lead. In contrast, for the lowest cortisol level (i.e. -1.5), higher testoterone level is increasing the probability to lead. Data on the order of arrival was collected by observations (a) and pup screams trials (b), and analyzed using mixed logistic models (GEE).

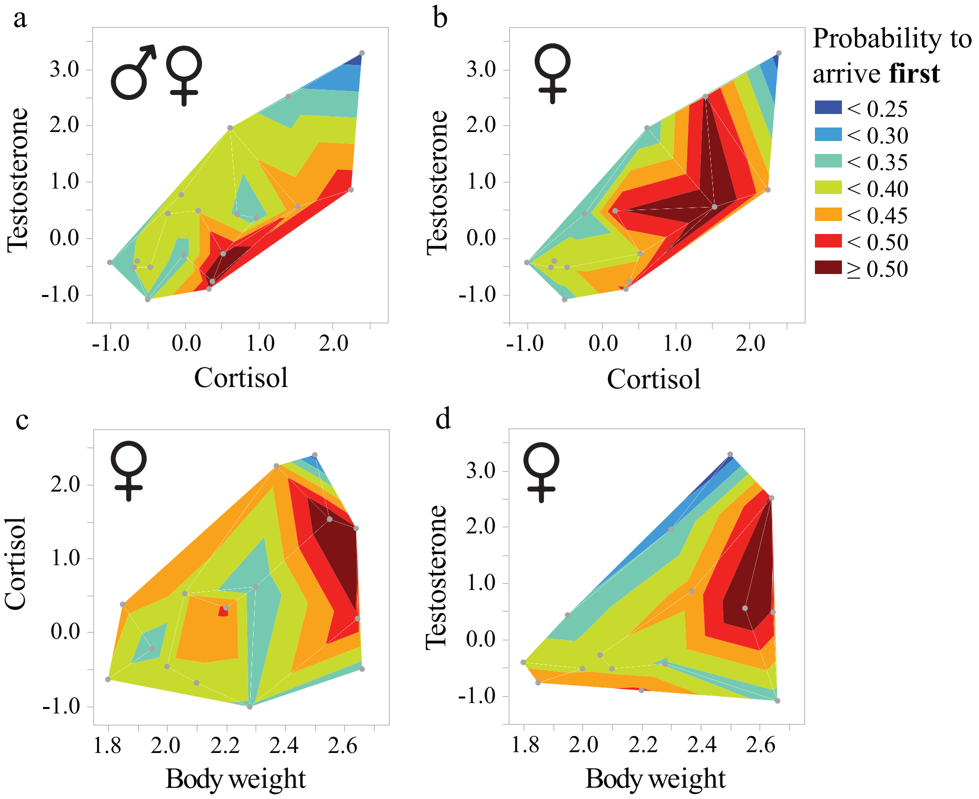

Fig. S5: **Coordinated movement sequences (low risk)**. Contour plots for the probability of arrival first to a base station as a function of hair cortisol and hair testosterone levels in both sexes combined (a), and in females only (b). The probability of order of arrival to a base station as a function of body weight and hair cortisol (c) and hair testosterone levels (d) in females only. The probability to arrive first is outlined by the colour keys on the right side of the figure. Hair cortisol and testosterone levels were standardized by year.

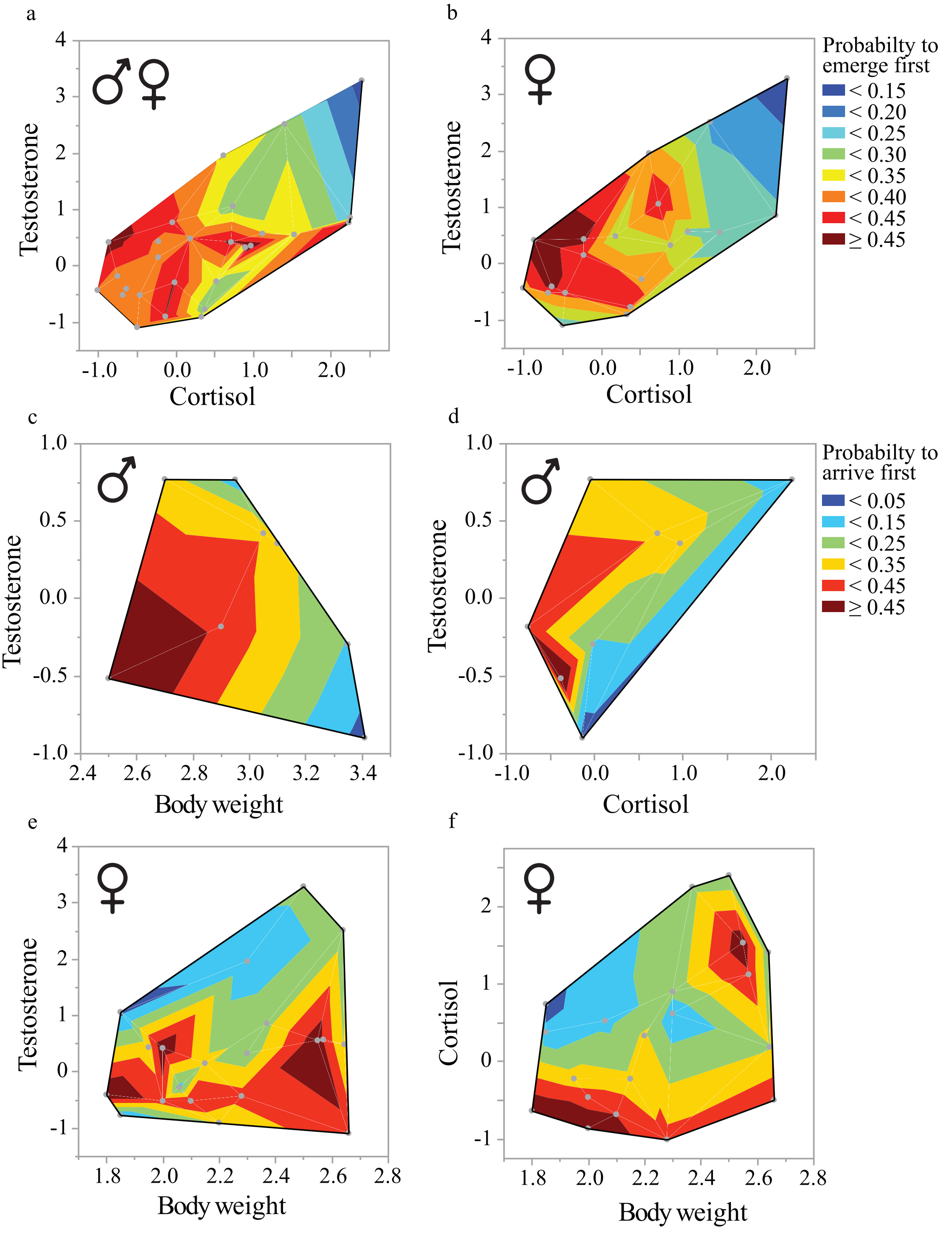

Fig. S6: **Coordinated movement and morning emergence sequences (low risk)**. Contour plots for the probability of first emergence from a burrow in the morning as a function of hair cortisol and hair testosterone levels in both sexes combined (a), and in females only (b). The probability of order of arrival first to a feeding tree as a function of body weight and hair testosterone (c) and hair cortisol and hair testosterone levels (d) in males only. The probability of order of arrival first to a feeding tree as a function of body weight and hair testosterone (e) and body weight and hair cortisol and levels (f) in females only. The probability to arrival first is outlined by the color keys on the right side of the figure. Hair cortisol and testosterone levels were standardized by year.

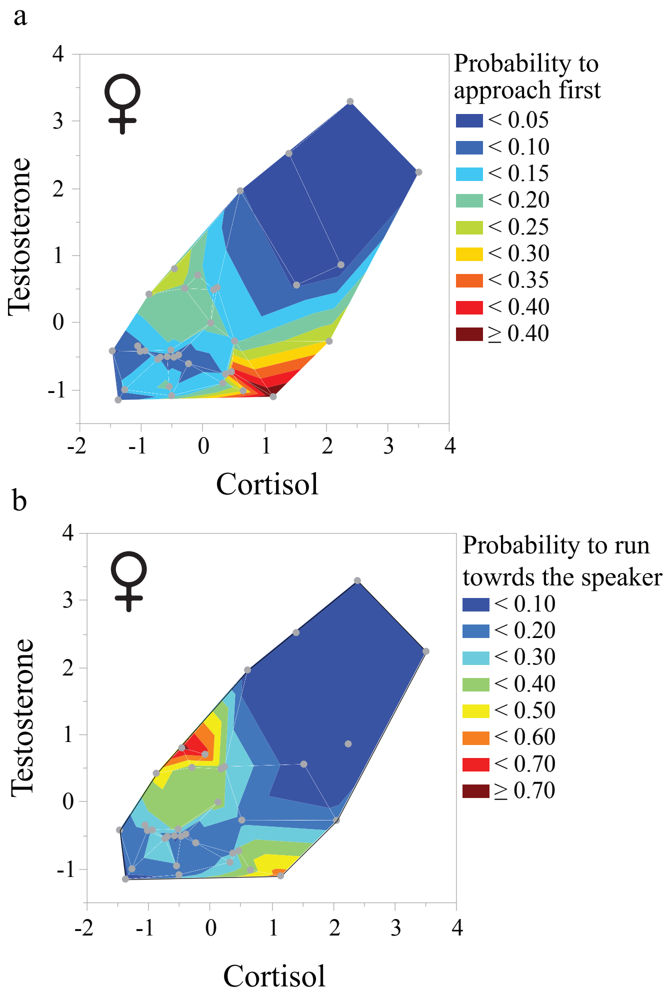

Fig. S7: **Predator defence events (high risk)**. Contour plots for the probability of females approaching first the speaker (a) and of females running towards the speaker (b). The probability to approach first or run towards the speaker is outline by the colour keys on the right side of the figure. Hair cortisol and testosterone levels were standardized by year.
